# Validation of the Arabic version of the diabetes self-management questionnaire in patients with type 2 diabetes mellitus: a cross-sectional study

**DOI:** 10.1186/s12875-024-02529-8

**Published:** 2024-07-29

**Authors:** Adnan Innab, Ali Kerari

**Affiliations:** Nursing Administration and Education Department, College of Nursing, King Saud University, Riyadh, 11421 Saudi Arabia

**Keywords:** DSMQ, Diabetes, Self-management, Reliability, Validation

## Abstract

**Background:**

The number of instruments available for measuring diabetes self-management activities in Arabic countries has been limited to date. To our knowledge, no multidimensional instrument suitable for measuring diabetes self-management is currently available in Arabic. This study assessed the validation of the Arabic version of the Diabetes Self-Management Questionnaire (A-DSMQ) in patients with type 2 diabetes mellitus (T2DM).

**Methods:**

This cross-sectional study was conducted from May to August 2022 at primary healthcare centers within the Riyadh region of Saudi Arabia. Four steps were followed during the translation and adaptation of the DSMQ: forward translation, consulting an expert panel, backward translation, and pilot testing on the target population. The data were collected using a convenience sample of 154 patients with T2DM. Cronbach’s α coefficient, criterion validity, and known-group validity were determined.

**Results:**

Cronbach’s α coefficient for internal consistency was 0.76. The A-DMSQ “sum scale” scores were negatively correlated with glycosylated hemoglobin (HbA1c) levels (Pearson’s *r* =  − 0.48, *p* < 0.01) and body mass indices (*r* =  − 0.29, *p* < 0.01) and positively correlated with Self-Rated Health Scale scores (*r* = 0.41, *p* < 0.01). Mean A-DSMQ “sum scale” scores differed significantly among groups with adequate, partially adequate, and inadequate glycemic control (*F* = 23.193, *p* < 0.001).

**Conclusions:**

These results indicate that the A-DSMQ is a reliable and valid tool for measuring diabetes self-management in patients with T2DM. The A-DSMQ can be used by researchers and healthcare providers interested in assessing diabetes self-management in this population. Healthcare providers should remain alert for suboptimal diabetes self-management, which may lead to significant economic costs in emergency and healthcare utilization.

## Highlights

What do we already know about this topic?

• Adequate diabetes self-management is essential for maintaining better glycemic control and achieving optimal health outcomes. However, evidence supports that many individuals living with diabetes report improvable self-management behaviors.

• The DSMQ is a widely used measure of self-management skills in areas of diabetes, which has been validated among individuals with T2DM in many countries.

How does your research contribute to the field?

• Our results provide additional evidence on the reliability and validity of the A-DSMQ in patients with T2DM.

• The A-DSMQ had appropriate structural and construct validity and acceptable internal consistency and reliability.

What are your research’s implications toward theory, practice, or policy?

• The A-DSMQ provides primary care nurses and other healthcare professionals with an easily administered tool for assessing the diabetes self-management skills of Arabic-speaking patients with T2DM.

## Background

Diabetes is a significant health problem worldwide, and its rate of diagnosis is currently soaring. Since 2015, diabetes has affected an estimated 340–536 million individuals worldwide [1]. Based on this estimate, the number of adults with type 2 diabetes mellitus (T2DM) will reach 829 million by 2040 [1]. While diabetes is considered widespread, only a third of patients achieve the ideal glycosylated hemoglobin (HbA1c) target, reflecting a controlled state. Uncontrolled diabetes contributes to morbidity and mortality, placing a heavy burden on healthcare systems worldwide [2, 3].

In Saudi Arabia, affluent lifestyles have increased among the population, with corresponding increases in obesity and related chronic diseases [4]. Diabetes affects about four million Saudi adults. Moreover, 1 in 10 of the remaining Saudi adults has a higher likelihood of developing diabetes, specifically prediabetes [3, 5]. Previous researchers have argued that the high prevalence of diabetes indicates poor self-management of a chronic illness by patients [6]. Therefore, the Ministry of Health and other government health agencies in Saudi Arabia have taken responsibility for implementing the diabetes management standards recommended by the American Diabetes Association. Accordingly, the role of Saudi hospitals and primary healthcare centers has been to systematically assess patients’ self-management practices related to diabetes.

HbA1c has been widely identified as a marker for predicting late-stage diabetes-related complications [7]. Glycemic control can minimize the risks of such complications. Moreover, adherence to diabetes self-management behaviors such as healthy dietary choices, regular physical activity, and blood glucose self-monitoring are imperative in establishing euglycemia. Therefore, patients’ self-management behaviors should be assessed to identify factors contributing to poor glycemic control. For this purpose, a validated self-management assessment instrument is valuable to researchers and healthcare professionals who want to examine multiple dimensions of diabetes self-management.

Several instruments have been used to assess self-management behaviors in various areas related to managing diabetes [8–15]. The available instruments were used to evaluate patients’ adherence to treatment plans or clarify their misconceptions about treatment plans [8, 13]. To our knowledge, no multidimensional instrument suitable for measuring diabetes self-management is currently available in Arabic.

A systematic review of measures for assessing patients’ adherence to diabetes self-management tasks identified 30 unique instruments [13], of which 21 addressed multiple dimensions of diabetes self-management behaviors [13]. However, only two instruments have been translated and validated in Arabic: the Summary of Diabetes Self-Care Activities Measure (SDSCA) and the Morisky Medication Adherence Scale. While the SDSCA and Morisky Medication Adherence Scale are reliable, valid, and available in Arabic, neither fully captures the self-management construct. To assess diabetes self-management behaviors among diverse groups of patients, researchers and healthcare providers must adapt and apply appropriate instruments after retesting and confirming their psychometric properties. Transcultural adaptation of existing validated tools can prove valuable for comparing studies on diabetes self-management.

Few instruments measuring diabetes self-management activities have been available in Arabic countries to date. For example, the Arabic version of the SDSCA is commonly used to assess diabetes self-management in Saudi Arabia. Several authors have stated that the SDSCA cannot robustly measure the association between self-management and glycemic control [16]. In addition, its short timeframe, considering only self-care activities reported by patients in the previous week, may influence its reliability in assessing proper adherence to diabetes self-management activities.

Compared to the SDSCA, the Diabetes Self-Management Questionnaire (DSMQ) is a relatively new psychometric tool with a broader time frame that may allow a more reliable assessment of self-management skills and an effective prediction of glycemic control [16, 17]. This instrument has been widely used to clinically evaluate patients presenting with inadequate diabetes self-management. In addition, it could be significant for studies examining the factors contributing to poor self-management and glycemic control in individuals with diabetes [16–18].

To our knowledge, while several researchers have translated the DSMQ into Arabic [19–23], none have tested its psychometric properties after translation or used it to assess self-management activities in patients with diabetes. Therefore, this study aimed to translate the DSMQ from English into Arabic and establish its validity in Middle Eastern populations using a sample of Saudi adults diagnosed with T2DM and currently visiting primary healthcare centers in Riyadh, Saudi Arabia.

## Methods

### Research design

This cross-sectional study was conducted from May to August 2022 at primary healthcare centers in the Riyadh region of Saudi Arabia. These primary healthcare centers provide healthcare services to patients with chronic diseases at various locations throughout the Riyadh region.

### Data collection procedure

Saudi adults with T2DM were recruited from primary healthcare centers in Riyadh, Saudi Arabia. Many adults with chronic diseases attend follow-up appointments at primary healthcare centers. Among adults who visited the centers, only those with T2DM were approached and recruited. The study’s inclusion criteria required participants to be (1) aged ≥ 18 years and (2) diagnosed with T2DM. Potential participants were excluded if they were pregnant, had cancer, or presented with cognitive disorders. Trained research assistants explained the study’s purpose and confidentiality to participants.

Participants completed the Arabic version of the DSMQ (A-DSMQ) scale at one point during their visits to the primary healthcare centers. The G*Power software was used to determine the required sample size. Considering a significance level of 0.05, a power of 0.80, and an effect size of 0.30, the minimum sample size was 111. This study’s sample comprised 154 patients, indicating an adequate sample size for the bivariate and multivariate analyses.

### Translation process

All study participants provided written informed consent. To develop a comprehensive and accepted translation, the researchers identified essential steps for the cross-cultural validation and adaptation of instruments [24]. They followed four steps during the translation and adaptation of the DSMQ: forward translation, consulting an expert panel, backward translation, and pilot testing on the target population.

In the first step, two professional bilingual translators translated the DSMQ from English into Arabic. In the second step, the translated A-DSMQ was presented to a panel of five experts knowledgeable about Arabic culture, who were asked to focus on the clarity, accuracy, and cultural relevance of the wording for each item, thereby establishing the foundation of the A-DSMQ. In the third step, an additional bilingual translator reverse-translated the A-DSMQ into English; the back translation of this instrument appropriately resembled the original DSMQ. In the fourth and final step, 15 participants with diabetes from primary healthcare centers pilot-tested the A-DSMQ. The selected participants provided feedback, indicating the items were clear and informative and confirming no misunderstandings when answering them. The final version of the A-DSMQ was established based on this translation process. Once this process was completed, the instrument was ready to be validated for use with Saudi individuals with diabetes.

### Measures

#### Demographic characteristics

The participants’ demographic characteristics were obtained by asking them six questions about their age, sex, education, body mass index (BMI), HbA1c, and time elapsed since diabetes diagnosis (in years). The participants’ demographic characteristics are presented in Table 1. The study sample comprised 154 patients with T2DM, with a response rate of 96.25%. Their mean age was 52 (standard deviation [SD]: ± 12.5), and their mean BMI was 26.8 (SD: ± 4.26). Slightly more participants were male (53%). Over half (57.5%) of the study participants had lived with T2DM for over five years. Their mean HbA1c level was 8.23 (SD: ± 2.03). Approximately 56% of the participants had HbA1c levels > 7.9%, indicating poor glycemic control according to the American Diabetes Association’s target criterion. Almost half of the study participants reported adequate diabetes self-management. The mean A-DSMQ score was 5.9 (SD: ± 1.29), with higher scores indicating adequate diabetes self-management.

**Table 1:** Characteristics of participants (*n* = 154)

**Variable**	**Mean ± SD or *n* (%)**
Age (years)	52 ± 12.5
Gender	
Male	80 (52)
Female	74 (48)
BMI	26.8 (4.26)
Time passed since diabetes diagnosis	
≤ 5 years	67 (42.5)
> 5 years	87 (57.5)
HbA1c	8.23 ± 2.03
DSMQ Sum Scores	5.91 ± 1.29
Self-rated Health Scale	3.10 ± 0.70

#### Diabetes self-management questionnaire

Developed by Schmitt et al. [17], the A-DSMQ was used to assess the study participants’ self-management skills related to their diabetes control status during the eight weeks preceding this study. A previous study reported that the DSMQ demonstrated adequate reliability and validity in German individuals with diabetes, with a Cronbach’s α of 0.84. The DSMQ comprises 16 items assessing four areas: glucose management (GM), dietary control (DC), physical activity (PA), and healthcare use (HU). Each item is rated on a four-point Likert-like scale from 0 (does not apply) to 3 (applies to me very much), giving a total DSMQ score between 0 (minimum) and 48 (maximum). In this study, the total DMSQ scores were transformed into a scale from 0 to 10, and adherence to diabetes self-management behaviors was categorized into three levels: inadequate (score of < 5), partially adequate (score of 5–8), and perfect (score of > 8).

#### Self-rated health scale

The Self-Rated Health Scale was used to measure the health status of the study participants. It was adapted from the U.S. National Health Survey and has been reported to be predictive of future health status [25]. The Self-Rated Health Scale comprises a single item rated on a five-point Likert-like scale from 1 (poor) to 5 (excellent); lower scores indicate worse health. The reliability of this scale was 0.92.

#### Glycemic control

The participants’ HbA1c levels were tracked to assess their glycemic control over three months. The research assistants recorded HbA1c levels from patients’ files concurrently with the psychometric assessments**.**

### Ethical considerations

This study was approved by the Institutional Review Board of the Saudi Ministry of Health before data collection began (approval number: H-01-R-012, IRB00010471; February 2022). The researchers informed each participant of the study’s purpose, its risks and benefits, and their rights to information privacy. The participants were informed that their information would be kept confidential and that they had the right to withdraw from the study without any consequences. The completed consent forms were obtained from those who met the inclusion criteria and decided to participate in this study.

### Data analysis

The data were managed and analyzed using the Statistical Package for the Social Sciences (version 28) and Analysis of a Moment Structures (version 28) software. Descriptive statistics are used to present the participants’ characteristics, while means and standard deviations are used to present the continuous variables.

The internal consistency of the A-DSMQ was assessed by computing Cronbach’s alpha coefficient for each subscale and the sum scale. A Cronbach alpha of > 0.70 is considered acceptable. In addition, item-total correlations and the effect of item removal on the coefficient were examined.

The construct validity of the A-DSMQ was evaluated by criterion validity and known-group validity. For criterion validity, Pearson’s product-moment correlation coefficient was used to determine correlations between diabetes self-management and variables theoretically or empirically related to diabetes self-management, such as self-rated health and HbA1c levels. A-DSMQ scores were expected to correlate (1) positively with self-rated health and (2) negatively with HbA1c levels. In addition, levels of diabetes self-management were expected to correlate negatively with BMI.

For known-group validity, a one-way analysis of variance was performed after categorizing the participants into three groups based on their HbA1c levels: HbA1c levels < 7% were classified as adequate glycemic control, from 7%– < 8% were classified as partially adequate glycemic control, and ≥ 8% were classified as inadequate glycemic control.

## Results

### Validation process

#### Internal consistency reliability

The Cronbach’s alpha for the A-DSMQ sum scale and its subscales were determined. The reliability analysis was conducted on the A-DSMQ sum scale comprising 16 items. It had a Cronbach’s alpha of 0.76, indicating acceptable internal consistency. The removal of any item did not increase Cronbach’s alpha. Correlation coefficients varied from 0.15 to 0.60 between items. Overall, the internal consistency results indicated that the A-DSMQ was reliable (Table 2).

**Table 2:** Item analyses and reliability of the A-DSMQ

Item	Mean (SD)	Corrected item-total correlation	Cronbach’s alpha if item deleted
1. Check blood sugar levels with care and attention	1.78 (0.86)	0.35	0.74
2. Choose food to easily achieve optimal blood sugar	1.70 (0.73)	0.31	0.75
3. Keep recommended doctors’ appointments	1.85 (0.90)	0.55	0.72
4. Take diabetes medication as prescribed	1.73 (1.02)	0.52	0.73
5. Occasionally eat lots of sweets/ high-carb foods	1.90 (0.90)	0.15	0.76
6. Record blood sugar levels regularly	1.22 (0.98)	0.25	0.75
7. Avoid diabetes-related doctors’ appointments	2.21 (0.92)	0.32	0.75
8. Do physical activity to achieve optimal sugar levels	1.37 (0.92)	0.16	0.76
9. Follow specialist’s dietary recommendations	1.38 (0.76)	0.17	0.76
10. Do not check blood sugar levels frequently enough	2.08 (0.87)	0.46	0.73
11. Avoid physical activity, although good for diabetes	1.94 (0.95)	0.46	0.73
12. Forget to take/ skip diabetes medication	2.03 (0.86)	0.60	0.72
13. Sometimes have real ‘food binges’	1.93 (0.81)	0.44	0.73
14. Should see medical practitioner(s) more often	1.50 (0.89)	0.20	0.76
15. Skip planned physical activity	1.74 (0.78)	0.36	0.74
16. Diabetes self-care is poor	2.01 (0.88)	0.54	0.72
Total			0.76

*A-DSMQ* Arabic Version of Diabetes Self-Management Questionnaire

The GM subscale, consisting of five items, had questionable reliability (α = 0.65). Item 12 (“I tend to forget or skip my diabetes medication”) showed a low item-total correlation (*r* = 0.18), and its removal increased Cronbach’s alpha (α = 0.69). The DC subscale, consisting of four items, had acceptable reliability (α = 0.71). The PA subscale, consisting of three items, had acceptable reliability (α = 0.72). Its three items appeared worthy of retention since their removal decreased Cronbach’s alpha. The HU subscale, consisting of three items, had poor reliability (α = 0.51).

#### Criterion validity

The correlations between A-DSMQ scores and variables of interest are shown in Table 3. A-DSMQ scores were significantly positively correlated with better self-rated health (*r* = 0.41, *p* < 0.01) and negatively correlated with HbA1c levels (*r* =  − 0.48, *p* < 0.01) and BMI (*r* =  − 0.29, *p* < 0.01). In other words, participants with normal body weight performed better in diabetes self-management.

**Table 3:** Correlations of DSMQ ‘Sum Scale’ and HbA1c, Self-rated Health Scale, and BMI

	Sum Scale
HbA1c	− 0.48**
Self-rated Health Scale	0.41**
BMI	− 0.29**

*DSMQ* Diabetes Self-Management Questionnaire, *HbA1c* glycated hemoglobin, *BMI* Body Mass Index

^**^p < .01

#### Known-groups validity

A-DSMQ scores differed significantly among participant groups stratified according to adequate, partially adequate, and inadequate glycemic control (*F* = 23.193, *p* < 0.001). These findings indicated that participants with adequate glycemic control (HbA1c < 7%) scored significantly higher on the A-DSMQ (6.97 ± 0.97) than those with partially adequate glycemic control (HbA1c of 7%– < 8%) and inadequate glycemic control (HbA1c of ≥ 8%). Notably, A-DSMQ scores did not differ significantly between the inadequate and partially adequate glycemic control groups (Table 4).

**Table 4:** Comparison of the DSMQ ‘sum scale’ in patients with HbA1c < 7%, from 7 to 8% and > 8%

DSMQ	HbA1c < 7%	Sign.^a^	HbA1c 7–8%	Sign.^b^	HbA1c > 8%	Sign.^c^	ANOVA
	(*n* = 49)		(*n* = 29)		(*n* = 76)		*P*-value
Sum Scale	6.97 ± 0.97	*	5.96 ± 1.18	ns	5.43 ± 1.09	***	< 0.001

Data are M ± SD. One-way ANOVA and Scheffé Test for post-hoc group comparisons were addressed. Scheffé Test significance is expressed

*DSMQ* Diabetes Self-Management Questionnaire, *HbA1c* glycated hemoglobin, *ANOVA* Analysis of Variance

^*^p < 0.05

^***^p < 0.001; ns, not significant

^a^regards comparison between the first and second group

^b^regards comparison between the second and third group

^c^regards comparison between the third and first group

## Discussion

The primary aim of this study was to create and validate the A-DSMQ using a convenience sample of patients with T2DM in Saudi Arabia. The A-DSMQ was created to provide a reliable and valid measure of diabetes self-management across medical settings. Since patients with poor diabetes self-management may constitute a high-risk group, a proper instrument may also prove valuable for clinical practice.

The original study recruited participants from a diabetes care center, with equal numbers of individuals with type 1 and 2 diabetes mellitus. In contrast, our study’s design may influence its generalizability since it only recruited participants with T2DM from primary healthcare centers. Indeed, there were noticeable differences in the populations between the original and our study. The HbA1c levels of primary care patients were lower in our study than in the original study (8.23 ± 2.03 vs. 8.6 ± 1.5). However, their DSMQ scores were also lower (5.91 ± 1.29 vs. 6.8 ± 1.7). Despite these differences, our study’s findings support the reliability and validity of the A-DSMQ.

In the context of Saudi patients with diabetes, the overall internal consistency (Cronbach’s alpha) of the A-DSMQ was found to be acceptable, albeit lower than in the study by Schmitt et al. Accordingly, the comparative analysis of the known groups supports the A-DSMQ as a valid tool for measuring self-management activities related to glycemic control. Furthermore, it was able to differentiate patients with varying levels of glycemic control.

The observed correlations between diabetes self-management and the variables expected to be associated with diabetes self-management (e.g., self-rated health and HbA1c level) were consistent with the study’s hypotheses, indicating criterion validity. A-DSMQ sum scores correlated significantly with glycemic control, assessed by HbA1c levels, and health status, assessed by the Self-Rated Health Scale. In addition, higher A-DSMQ sum scores were significantly associated with optimal glycemic control (HbA1c ≤ 7.5%) and excellent health status. These findings support the notion that assisting patients in adopting self-management tasks (e.g., dietary control, physical activity, and glucose management) may lead to proper glycemic control, optimal health status, and reduced diabetes-related complications [6, 26]. These correlations were generally stronger than those found in a Hungarian study of the DSMQ, in which a minimal association (*r* = 0.25) was reported between DSMQ sum scores and HbA1c levels [18]. In another study that evaluated diabetes self-management skills among Iranian patients with T2DM, lower disease-related complications were reported among those who scored higher on the DSMQ [27].

### Implications and recommendations for research and practice

The results revealed that A-DSMQ had very good psychometric properties. Thus, the Arabic version of this instrument can be used among Arab adults with type 2 diabetes. Future researchers are recommended to re-test the A-DSMQ across a wide-nation of Arab adults to determine the generalizability of the findings to other settings. It is also recommended to use a probability sampling method to assure that the results are unbiased and generalizable to other settings. Other essential variables, such as health literacy, should be taken into consideration to better understand the applicability of using the Arabic version of the instrument for patients with type 2 diabetes.

### Limitations

This study is the first to validate the DSMQ in Arabic. However, it had several limitations. Firstly, it used a cross-sectional design, so it could not infer causality. Secondly, it used a convenience sampling method, so its findings might be less applicable to the primary care setting. Thirdly, it did not include patients with type 1 diabetes, so its results are limited to patients with T2DM. Fourthly, it only registered HbA1c levels over three months, but individuals’ behavior can change within a shorter period. A more appropriate method would have involved performing HbA1c blood sampling concurrently with administering psychometric assessments, which was not implemented due to financial limitations.

## Conclusions

The A-DSMQ was found to be a reliable and valid instrument for measuring self-management behaviors in patients with T2DM in Saudi Arabia. Our findings for the A-DSMQ are consistent with those of the original study on the DSMQ [17]. Our results indicated a significant and negative relationship between diabetes self-management behaviors and glycemic control, indicating an adequate known-groups validity. Therefore, this instrument could be used to predict HbA1c levels. In addition, the A-DSMQ may be valuable for clinical use, where it can assist healthcare providers in detecting poor diabetes self-management. The A-DSMQ could be an outcome measure for evaluating the effectiveness of self-management interventions among patients with T2DM. The accessibility and availability of the A-DSMQ may also inspire future research in the field.

## Data Availability

The datasets used and analyzed during the current study available from the corresponding author upon reasonable request.
